# Protective Effects of Cirsilineol against Lipopolysaccharide-Induced Inflammation; Insights into HO-1, COX-2, and iNOS Modulation

**DOI:** 10.3390/ijms24108537

**Published:** 2023-05-10

**Authors:** Go Oun Kim, Dong Ho Park, Jong-Sup Bae

**Affiliations:** 1 Research Institute of Pharmaceutical Sciences, College of Pharmacy, Kyungpook National University, Daegu 41566, Republic of Korea; 2 Department of Ophthalmology, School of Medicine, Kyungpook National University, Kyungpook National University Hospital, Daegu 41944, Republic of Korea

**Keywords:** cirsilineol, endothelium, iNOS, p-STAT-1

## Abstract

In this study, the potential protective effects of cirsilineol (CSL), a natural compound found in *Artemisia vestita*, were examined on lipopolysaccharide (LPS)-induced inflammatory responses. CSL was found to have antioxidant, anticancer, and antibacterial properties, and was lethal to many cancer cells. We assessed the effects of CSL on heme oxygenase (HO)-1, cyclooxygenase (COX)-2, and inducible nitric oxide synthase (iNOS) in LPS-activated human umbilical vein endothelial cells (HUVECs). We also examined the effects of CSL on the expression of iNOS, tumor necrosis factor (TNF)-α, and interleukin (IL)-1β in the pulmonary histological status of LPS-injected mice. The results showed that CSL increased HO-1 production, inhibited luciferase-NF-κB interaction, and reduced COX-2/PGE2 and iNOS/NO levels, leading to a decrease in signal transducer and activator of transcription (STAT)-1 phosphorylation. CSL also enhanced the nuclear translocation of Nrf2, elevated the binding activity between Nrf2 and antioxidant response elements (AREs), and reduced IL-1β expression in LPS-treated HUVECs. We found that CSL’s suppression of iNOS/NO synthesis was restored by inhibiting HO-1 through RNAi. In the animal model, CSL significantly decreased iNOS expression in the pulmonary biostructure, and TNF-α level in the bronchoalveolar lavage fluid. These findings indicate that CSL has anti-inflammatory properties by controlling iNOS through inhibition of both NF-κB expression and p-STAT-1. Therefore, CSL may have potential as a candidate for developing new clinical substances to treat pathological inflammation.

## 1. Introduction

Heme oxygenase-1 (HO-1) plays a significant role in protecting against inflammation-induced damage and the accumulation of reactive oxygen species (ROS), particularly in lethal illnesses such as acute autoimmune response, lung conditions, and malignant tumors [1,2]. HO-1 inhibits the synthesis of proinflammatory cytokines and factors such as tumor necrosis factor (TNF)-α, interleukin (IL)-1β, and IL-6 [2]. Previous studies have shown that HO-1 has a promising impact on protecting mice from acute septic inflammation induced by cecal ligation and perforation, and also on a number of vascular illnesses related to inflammation [2,3]. The Kelch-like ECH-associated protein 1 (Keap1)-Nrf2-antioxidant response element (ARE) pathway plays a principal role in the adjustment of oxidative stress and homeostasis of the reverse inflammatory reaction by regulating not only antioxidant genes, but also detoxification genes for operating and degrading cancer-causing agents and toxic elements [4]. Nrf2 acts as a major factor in the modulation of cytoprotective action in response to extracellular conditions. The Nrf2-ARE pathway is recognized as a key target for the clinical approach of inflammation-mediated disorders [4,5]. The signal transducer and activator of transcription (STAT)-1 and nuclear factor (NF)-κB are two important signaling pathways that play a critical role in inflammation [6,7]. The activation of STAT-1 and NF-κB leads to the production of proinflammatory cytokines, chemokines, and adhesion molecules, which recruit immune cells to the site of inflammation [6,7]. However, the dysregulation of these pathways can lead to chronic inflammation, tissue damage, and the development of various diseases such as cancer, autoimmune disorders, and metabolic disorders [6,7]. Therefore, targeting STAT-1 and NF-κB signaling pathways represents a promising therapeutic approach for the treatment of inflammation-related diseases.

Lung functional disorder is a lethal respiratory failure caused by inflammation, characterized by proliferative edema, blood hypoxia, and neutrophil extracellular traps inside the lung. Lipopolysaccharide (LPS) is the main pathogenic factor of lung disorders, promoting the expressions of proinflammatory mediators through the activation of transcriptional proteins [8]. These inflammation-mediated molecules not only upregulate long-term inflammation, but also cause the development of inflammatory disorders such as vascular dysfunction, asthma, chronic obstructive pulmonary disease (COPD), and cystic fibrosis [8].

Traditional Chinese herbal remedies have been used to cure several conditions, including diabetes, inflammatory disorders, liver disease, stroke, and cardiovascular disease [9,10]. However, scientific research does not support these findings [9,10]. The flavone bioactive substance cirsilineol (CSL), found in the Chinese and Tibetan plant *Artemisia vestita*, is a powerful anti-inflammatory, hypnotic, anticancer, antibacterial, and anti-anxiety medication that demonstrates cytotoxicity against several cancer cells [11,12,13,14]. The activation network of how CSL suppresses HO-1 and other inflammatory mediators, such as TNF-α, IL-1β, and NO, in HUVECs in vitro or histologic structures of LPS-injected mice in vivo are unidentified. This study aims to prove and define the role of CSL on the triggering of HO-1 signal transduction and inhibition of inflammatory cytokines, and to discover the prominent subprocess of how CSL works as a potential substance targeting inflammatory pathology.

## 2. Results and Discussion

### 2.1. Effect of CSL on the Levels of iNOS and COX-2 in LPS-Activated HUVECs

**Figure 1:**
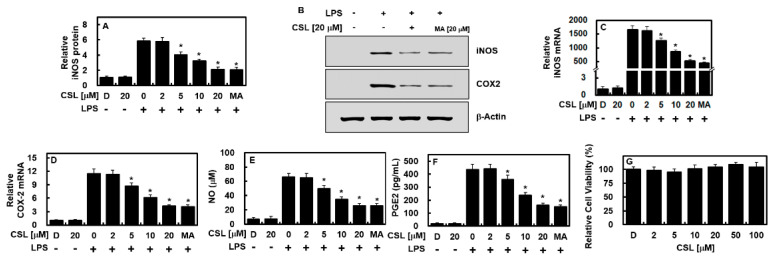
CSL reduced the levels of COX-2 and iNOS in HUVECs treated with LPS. Following stimulation with LPS (1 μg/mL) for 6 h, HUVECs were treated with different concentrations of CSL or MA (20 μM) for another 6 h, and the levels of iNOS protein (**A**,**B**), COX-2 protein (**B**), iNOS mRNA (**C**), COX-2 mRNA (**D**), NO (**E**), and PGE2 (**F**) were measured. The results presented are the mean ± standard deviation (SD) values from three independent experiments, each conducted in triplicate on different days. The effect of CSL on cellular viability was evaluated using the MTT assay (**G**). D refers to treatment with 0.2% DMSO, which was used as the vehicle control. * *p* < 0.05 compared to LPS.

To investigate the effects of CSL on inflammation-related gene expressions, the expressions of iNOS and COX-2, two well-known proinflammatory mediators, were measured. Maslinic acid, a natural compound of the triterpenoid group derived from olive, was used as a positive control [15]. After 6 h of LPS treatment, HUVECs were treated with different doses of CSL or 20 μM of MA for another 6 h. The results of qPCR, ELISA, and immunoblot assays showed that the expressions of iNOS and COX-2 induced by LPS were downregulated, in a dose-dependent manner, by CSL treatment or by MA at 20 μM (Figure 1A–D). The expressions of their related molecules, PGE2 and NO, were also decreased after CSL or MA treatment (Figure 1E,F). To determine the possible cytotoxic effects of CSL on HUVECs, the MTT assay was performed, and the results showed no significant change in cell viability up to 100 μM of CSL (Figure 1G). These findings indicate that CSL effectively inhibited iNOS production and prevented LPS-induced NO synthesis.

### 2.2. Effect of CSL on NF-κB Activity, STAT-1 Phosphorylation, and HO-1 Protein Level in LPS-Activated HUVECs

**Figure 2:**
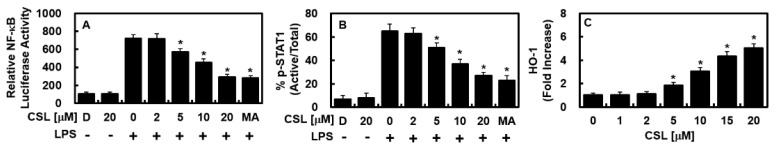
CSL inhibited the activities of NF-κB and STAT-1, and upregulated the protein level of HO-1. Following LPS stimulation of HUVECs, CSL was administered at indicated concentrations or MA (20 μM) for 6 h. The protein level of HO-1 (**C**) was upregulated by CSL, and NF-κB activity (**A**) and STAT-1 phosphorylation (**B**) were suppressed. (**A**) NF-κB activity was measured in cells that were transfected with the NF-κB luciferase reporter vector, (**B**) while the phosphorylation of STAT-1 was measured by ELISA. (**C**) HO-1 expression from extracted proteins was also analyzed by ELISA. The results are presented as the mean ± SD from three independent experiments conducted in triplicate on three different days. DMSO treatment (0.2%) was used as the vehicle control, denoted as D. * *p* < 0.05 compared to LPS.

Next, we examined the effect of CSL on the regulation of NF-κB, which is known to play a significant role in inflammation-related gene expression. Results showed that CSL had a dose-dependent inhibitory effect on NF-κB luciferase reporter response, as depicted in Figure 2A. It was previously reported that the JAK/STAT signaling pathway is a key regulatory mechanism in the production of iNOS and COX-2 during LPS treatment [16,17]. Thus, this study investigated the effect of CSL on STAT-1 phosphorylation and its product. Results revealed that CSL not only suppressed STAT-1 phosphorylation and its product, as shown in Figure 2B, but also significantly increased the level of HO-1, as shown in Figure 2C.

### 2.3. Effects of CSL on the Nuclear Translocation Activity of Nrf2, ARE Reporter, and Anti-Inflammatory Activity

**Figure 3:**
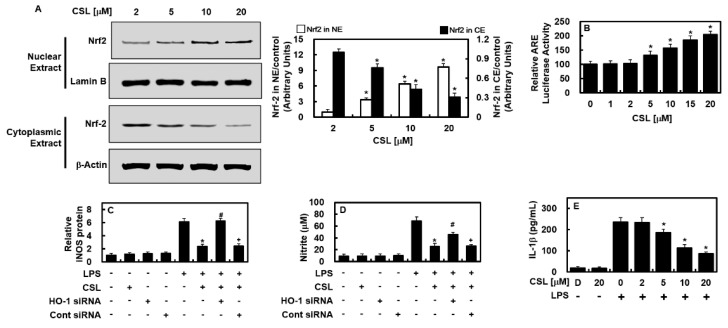
In HUVECs, CSL caused the translocation of Nrf2 to the nucleus and had an anti-inflammatory effect. (**A**) HUVECs were treated with CSL (2–20 μM) for 6 h, and cytosolic and nuclear fractions were extracted. The densitometric intensity of Nrf2 was determined using Western blotting, and (**B**) ARE luciferase reporter activity was measured using lysates from cells transfected with ARE. (**C**–**E**) To determine whether CSL-mediated HO-1 expression was responsible for iNOS and NO inhibition, HO-1 expression was suppressed using siRNA. IL-1β concentrations were measured using an ELISA kit. The results are presented as the mean ± SD from three independent experiments conducted in triplicate on three different days. DMSO treatment was used as a vehicle control, denoted as D. LPS was used as a reference for comparison, and * *p* < 0.05 vs. LPS, ^#^ *p* < 0.05 vs. LPS + CSL, or ^+^ *p* < 0.05 vs. LPS + CSL + HO-1 siRNA.

Given that the expression of HO-1 and other antioxidative proteins relies on Nrf2, we examined how CSL affects Nrf2 nuclear localization and ARE expression. The results indicated that CSL increased Nrf2 nuclear translocation and upregulated ARE luciferase reporter response (Figure 3A,B). We investigated whether the suppression of iNOS expression by CSL was due to HO-1 production by using small interference RNA (siRNA) to suppress HO-1. The suppression of HO-1 led to the recovery of iNOS and NO expressions similar to those in cells without CSL treatment, indicating that CSL enhances HO-1 expression by reducing iNOS levels (Figure 3C,D). Furthermore, CSL’s ability to alleviate inflammation was demonstrated by the downregulation of IL-1β expression in LPS-treated HUVECs (Figure 3E).

### 2.4. Effect of CSL on TNF-α and iNOS Protein Levels in the LPS-Mediated Lung Injury Mouse Model

**Figure 4:**
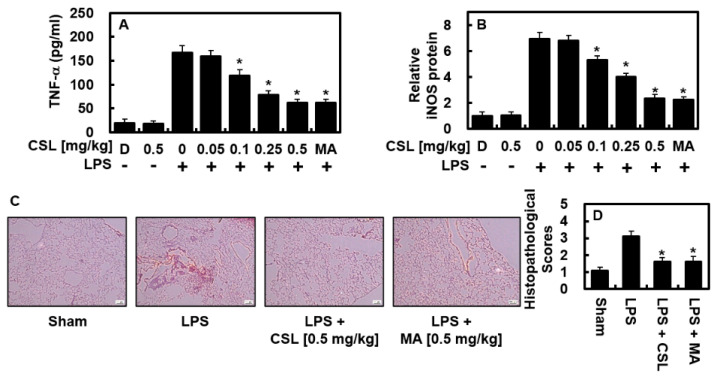
In LPS-injected mice, CSL suppressed the levels of TNF-α and iNOS, and alleviated lung tissue injury. The experiment started by injecting LPS (15 mg/kg, i.p.), followed by the administration of CSL (0.05–0.5 mg/kg, i.v.) or MA (0.5 mg/kg) after 6 h. The control group was not injected with LPS. Five mice were used for each group. The lung tissue and BALF were collected one day after the LPS injection, and the levels of TNF-α (**A**) and iNOS (**B**) were analyzed. The results were obtained from three independent experiments conducted in triplicate on three different days, and the mean ± SD values were reported. D represents 0.2% DMSO, which served as the vehicle control. Lung tissue samples were stained with H&E, and representative images from three independent experiments were shown (**C**). Scale bar: 200 μm. The histopathological scores for the lung tissue were recorded (**D**) as described in the Methods section. * *p* < 0.05 vs. LPS.

We examined the anti-inflammatory effects of CSL in animal models. As shown in Figure 4A, the elevated TNF-α production induced by LPS was markedly reduced in the BALF of mice treated with CSL or MA (0.5 mg/kg). Considering the expected blood volume of mice (72 mL/kg) [18,19] and the body weight of the mice used in this study (27 g), the CSL treatments of 0.05, 0.1, 0.25, and 0.5 mg/kg were converted to calculated concentrations of up to 2, 5, 10, and 20 μM, respectively, in the peripheral fluid. Similarly, MA at 0.5 mg/kg was calculated to be 20 μM in the peripheral fluid. In the lung tissue, the expression of iNOS was significantly reduced after the injection of CSL or MA (Figure 4B), indicating the anti-inflammatory activity of CSL in vivo. The histological observations (Figure 4C,D) further demonstrated that CSL or MA effectively ameliorated LPS-induced pulmonary damage.

This study found that LPS stimulation led to increased levels of inflammatory mediators (NO, PGE2, TNF-α, and IL-1β) and regulatory enzymes (iNOS and COX-2), but the increase was inhibited by CSL, indicating that CSL has anti-inflammatory and antioxidant properties in both LPS-stimulated cells and mice. When inflammation occurs, activated endothelial cells release inflammatory mediators (NO, PGE2, TNF-α, IL-1β) and regulatory enzymes (iNOS and COX-2) [20,21]. However, CSL significantly decreased the levels of these inflammatory mediators and regulatory enzymes in LPS-stimulated cells and mice, indicating its anti-inflammatory and antioxidant effects. In addition, we showed that CSL increased the expression of HO-1 in a dose-dependent manner, and it also reduced the levels of COX-2/PGE2 and iNOS/NO induced by LPS, as well as the activity of NF-κB. NF-κB plays a crucial role in various inflammatory processes, including cell adhesion, proliferation, differentiation, and apoptosis alleviation [22]. Furthermore, NF-κB also regulates immune responses by activating proinflammatory signaling during inflammation. An increase in NO is linked to inflammatory responses in the airway by regulating chemokine production, and adequate activation of NF-κB is required for LPS-induced expression of COX-2 and iNOS. This study suggests that CSL suppresses HO-1 production and the expression of proinflammatory mediators (iNOS, COX-2, IL-1β, and NO) by modulating NF-κB activity.

Furthermore, HO-1 production induced by CSL may have led to the suppression of iNOS and TNF-α expression in the BALF of LPS-administered mice. Our results from both current and previous studies suggest that CSL induces HO-1, which can inhibit NF-κB activation and/or oxidative enzyme activity, leading to a reduction in substrate availability for COX-2 and STAT-1 phosphorylation. Additionally, CSL upregulated the ARE luciferase reporter activity and affected the nuclear translocation of Nrf2. Therefore, the anti-inflammatory effects of CSL can be attributed to the upregulation of HO-1 expression and downregulation of iNOS expression. These findings were supported by RNAi-mediated HO-1 inhibition, which reversed the CSL-mediated inhibition of NO production and iNOS expression. Thus, our study shows that CSL increased HO-1 expression and reduced the production of proinflammatory mediators in LPS-treated HUVECs and LPS-injected mice lung tissues, including iNOS and TNF-α levels. These results suggest the importance of HO-1 in suppressing inflammatory responses, with TNF-α potentially playing a crucial role. Our study also demonstrates that CSL can modulate inflammation by regulating the levels of HO-1 expression, leading to the inhibition of oxidase response and/or NF-κB activation. CSL can induce HO-1 expression in a dose-dependent manner, while also reducing the levels of NF-κB expression. Previous studies have shown that activation of the Nrf2 antioxidant pathway can prevent the transcriptional upregulation of proinflammatory cytokines induced by LPS [4]. Given that CSL was found to significantly inhibit proinflammatory cytokines (TNF-α and IL-1β), it was hypothesized that the anti-inflammatory effect of CSL may be mediated through activation of the Nrf2 pathway. To test this hypothesis, this study examined the expression of Nrf2 and Keap1 in LPS-stimulated cells, and found that CSL dose-dependently triggered the nuclear translocation of Nrf2 and downregulated the level of Keap1. These results suggest that CSL may exert its anti-inflammatory and antioxidant effects by activating the Nrf2 antioxidant pathway.

The anti-inflammatory effects of CSL in vivo have been extensively studied, and have shown promising results. This study examined the effects of CSL on TNF-α and iNOS protein levels in a mouse model of LPS-mediated lung injury. This study found that CSL treatment significantly reduced the production of TNF-α in the BALF of mice, indicating that CSL has potent anti-inflammatory effects. Moreover, the expression of iNOS in the lung tissue was significantly reduced after treatment with CSL, further supporting its anti-inflammatory activity in vivo. In addition to these findings, this study also showed that CSL effectively ameliorated LPS-induced pulmonary damage. This suggests that CSL may have a protective effect on lung tissue during inflammation. Overall, these results highlight the potential of CSL as a therapeutic strategy for treating inflammatory pathologies, particularly those related to the respiratory system. It is important to note that the doses of CSL used in this study were converted to calculated concentrations of up to 2, 5, 10, and 20 μM in the peripheral fluid. This provides useful information for future studies, and helps to establish a basis for determining appropriate dosages of CSL for therapeutic use in humans. Overall, the results of this study demonstrate the anti-inflammatory effects of CSL in vivo, particularly in the context of lung inflammation. This information could be useful in the development of new therapies for the treatment of various inflammatory disorders. However, further studies are needed to fully understand the mechanisms underlying the anti-inflammatory effects of CSL, and to determine its potential for clinical use.

**Figure 5:**
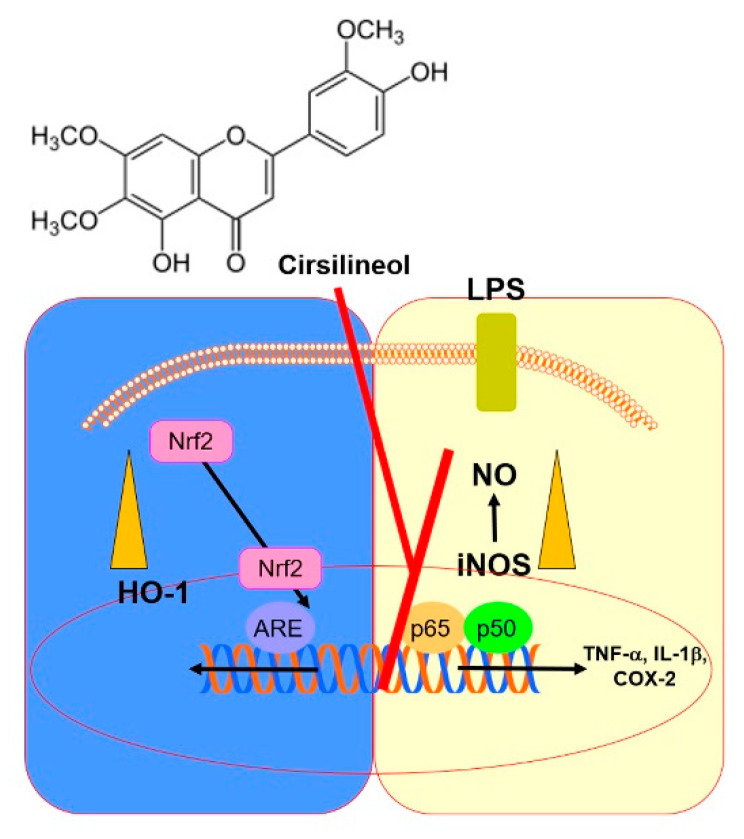
A scheme summarizing the research conclusion.

In summary, this study demonstrates that CSL effectively increases the level of HO-1 and suppresses the synthesis of proinflammatory cytokines in HUVECs treated with LPS. It also reduces the expression of iNOS and TNF-α in lung tissues from LPS-treated mice. These results highlight the importance of HO-1 in regulating inflammatory responses, and suggest that TNF-α mediates the HO-1 pathway (Figure 5). Therefore, CSL may be a promising therapeutic strategy for treating inflammatory pathologies, especially those related to the respiratory system.

## 3. Materials and Methods

### 3.1. Cell Culture and Reagents

Human umbilical vein endothelial cells (HUVECs) were used, which were obtained from Cambrex BioScience (C2517AT25; Charles City, IA, USA) and managed according to the same protocol used in previous research [23]. The following reagents were commercially obtained from Sigma Chemical Co. (St. Louis, MO, USA): CSL, MA, LPS (L2654; isolated from *Escherichia coli*), penicillin G, streptomycin, and dimethyl sulfoxide (DMSO). Human HO-1 (sc-35554) or control siRNA (sc-37007) were manufactured by Santa Cruz Biotechnology (Santa Cruz, CA, USA). HUVECs that had been subcultured for 3–5 passages were seeded in a dish (density, 1 × 10^5^ cells per 35 mm diameter) and made nutrient-deficient overnight for the enzyme-linked immunosorbent assay (ELISA). Some cells were treated with LPS (1 μg/mL for 6 h) and then treated with CSL for 6 h, while other cells were treated with CSL for 6 h without being treated with LPS (to measure the level of HO-1).

### 3.2. LPS-Injected Lung Injury Mouse Model

Male C57BL/6 mice, aged between 6 and 7 weeks and weighing an average of 27 g, were procured from Orient Bio Inc. (Seongnam, Republic of Korea). Before using the mice for each experiment, they were given a 12-day acclimatization period for adjustment, as previously described [23,24]. Intraperitoneal injection of LPS (15 mg/kg) with 0.2% DMSO (vehicle control) was performed using 28-gauge needles. At 6 h after the administration, intravenous injection of CSL (0.05–0.5 mg/kg) was performed. The Animal Care Committee at Kyungpook National University approved the method (IRB No. KNU 2022-107). Moderate suction was used to obtain bronchoalveolar lavage fluid (BALF) after intratracheal administration of phosphate-buffered saline (PBS), followed by centrifugation at 3000 rpm for 10 min at 4 °C. The final supernatant was stored in a freezer at −80 °C for subsequent investigations.

### 3.3. ELISA

The HUVECs were subjected to LPS stimulation (1 μg/mL, 6 h) and then exposed to CSL for 6 h. For the detection of HO-1 concentration, CSL was applied to the remaining cells for 6 h without LPS activation. The levels of STAT-1 phosphorylation were evaluated using ELISA kits from Abcam (ab126455, Cambridge, MA, USA). To measure the levels of PGE2, HO-1, IL-1β, TNF-α, and iNOS, ELISA kits from R&D Systems were used, and the upper fluid of the cell culture after centrifugation was utilized for the same.

### 3.4. Cell Viability Assay

To assess the cell viability, the researchers used the 3-(4,5-dimethylthiazol-2-yl)-2,5-diphenyltetrazolium bromide (MTT) assay method, which was described in previous studies [23,24,25]. HUVECs were seeded at a concentration of 5 × 10^3^ cells/well in 96-well plates and treated with CSL for 48 h. The cells were then washed and incubated for an additional 4 h after adding 100 µL of MTT (1 mg/mL) to each well. The formazan salt formed was dissolved by adding 150 µL of dimethyl sulfoxide (DMSO), and the level of formazan salt was measured at a wavelength of 540 nm using a spectrometer (Tecan, Austria GmbH, Grödig, Austria). The cell viability with treatment was expressed as a percentage of the viability of untreated cells, which was considered as 100%.

### 3.5. Cell Viability Assay Nitrite Levels

The amount of nitric oxide produced was measured by calculating the concentration of nitrite (NO_2_^−^) present in the media of the cells. To do this, an equal amount of Griess reagent (ab234044, Abcam) was added to the supernatant and left in an incubator for 15 min at room temperature. The resulting solution was then analyzed using a spectrometer (λ = 540 nm). The measurements were repeated three times to ensure accuracy.

### 3.6. Intracellular Fractionation and Immunoblotting

The cells were obtained and the supernatant was separated using a centrifuge. The cytosolic and nuclear extracts were prepared following a previous protocol [26]. Immunoblotting was performed using antibodies against iNOS, COX-2, lamin B, Nrf2, and β-actin (Santa Cruz, CA, USA). Lamin B and β-actin served as loading controls for the cytosolic and nuclear extracts, respectively.

### 3.7. Quantitative Real-Time Polymerase Chain Reaction

TRI reagent (Invitrogen, Waltham, MA, USA) was used to extract RNA, which was then reverse-transcribed in a 20 µL reaction mixture using 0.5 mg/µL of the oligo (dT)-adapter primer (Invitrogen) and M-MLV reverse transcriptase (Invitrogen) with the PX2 Thermal Cycler (Thermo Scientific, Waltham, MA, USA). The expressions of iNOS and COX-2 were compared with those of β-actin, and the following primer sequences were designed for qRT-PCR analysis: COX-2 forward: 5′-CCC CAT TAG CAG CCA GTT-3′, COX-2 reverse: 5′-CAT TCC CCA CGG TTT TGA-3′; iNOS forward: 5′-GTT CTC AGC CCA ACA ATA CAA GA-3′, iNOS reverse: 5′-GTG GAC GGG TCG ATG TCA C-3′; and β-actin forward: 5′-TCGTGCGTGACATCAAAGA-3′, β-actin reverse: 5′-CAT ACC CAA GAA GGA AGG CT-3′.

### 3.8. Transfection

The NF-κB luciferase reporter vector, HO-1 siRNA, ARE luciferase reporter vector, and nontranscript control siRNA were prepared using SuperFect (Qiagen, Valencia, CA, USA) and used for plasmid transfections. The plasmid-transfected cells were incubated for 4 h, after which the medium was replaced with fresh medium.

### 3.9. ARE Luciferase Reporter Assay

The cells were washed gently with mild temperature PBS and then lysed using lysis buffer from the dual luciferase kit (Promega, Madison, WI, USA). The resulting luciferase activity was measured using a TD-20/20 luminometer (Turner Designs, San Jose, CA, USA). All transfections were carried out three times independently. The results were expressed as the ratio of the luciferase activity of firefly to that of *Renilla*.

### 3.10. Histopathological Analysis

Intraperitoneal injection of LPS was administered to five mice, followed by treatment with CSL (0.5 mg/kg, i.v.) after 6 h. The mice were euthanized and sacrificed, and histological differences in the lung tissue samples were examined using hematoxylin and eosin (H&E) staining, as per previous research [25]. The pulmonary structure scores were graded from 1 to 4 based on the established grading system [26].

### 3.11. Statistical Analysis

The figures presented in this study are the mean values ± standard deviation (SD) of three independent trials. To determine significant differences between groups, one-way analysis of variance and Tukey’s post hoc test were used. Statistical significance was set at a *p*-value of less than 0.05.

## Data Availability

The data presented in this study are available upon reasonable request from the corresponding author.
